# CLASH—A Compliant Sensorized Hand for Handling Delicate Objects

**DOI:** 10.3389/frobt.2019.00138

**Published:** 2020-01-17

**Authors:** Werner Friedl, Máximo A. Roa

**Affiliations:** German Aerospace Center-DLR, Institute of Robotics and Mechatronics, Wessling, Germany

**Keywords:** hand design, end effector, soft manipulation, variable impedance, grasp stiffness

## Abstract

Automation of logistic tasks, such as object picking and placing, is currently one of the most active areas of research in robotics. Handling delicate objects, such as fruits and vegetables, both in warehouses and in plantations, is a big challenge due to the delicacy and precision required for the task. This paper presents the CLASH hand, a Compliant Low-Cost Antagonistic Servo Hand, whose kinematics was specifically designed for handling groceries. The main feature of the hand is its variable stiffness, which allows it to withstand collisions with the environment and also to adapt the passive stiffness to the object weight while relying on a modular design using off-the-shelf low-cost components. Due to the implementation of differentially coupled flexors, the hand can be actuated like an underactuated hand but can also be driven with different stiffness levels to planned grasp poses, i.e., it can serve for both model-based grasp planning and for underactuated or model-free grasping. The hand also includes self-checking and logging processes, which enable more robust performance during grasping actions. This paper presents key aspects of the hand design, examines the robustness of the hand in impact tests, and uses a standardized fruit benchmarking test to verify the behavior of the hand when different actuator and sensor failures occur and are compensated for autonomously by the hand.

## 1. Introduction

Soft manipulation is a hot topic of research in robotics nowadays, as it promises a simpler way to bring multi-fingered hands to real-world scenarios. Indeed, soft hands are intrinsically robust for interacting with the environment and require simple control strategies to actuate a reduced (mostly one) number of actuated degrees of freedom (DoF) (Dollar and Howe, 2010). Applications in the logistics and food processing industries are one of the potential scenarios where soft robotic hands could automate the grasping of different objects with very different weights and shapes. Inspired by observation of human grasping actions, the use of environmental constraints (Eppner et al., 2015) increases the performance of soft hands by reducing the influence of uncertainty coming from vision and sensors and proves valuable in scenarios, such as fruit handling, where there is no CAD model available to describe the shape of the manipulated objects. Many different soft robotics hand technologies have been developed in recent years, mostly with embodied compliance (Aukes et al., 2014; Catalano et al., 2014; Ciocarlie et al., 2014; Stuart et al., 2017). For example, the hand presented in Tavakoli and de Almeida (2014) uses structural elasticity (elastic joints and soft pads), and the hand from Deimel and Brock (2015) uses structural elasticity paired with differential actuation. However, pneumatic hands with structural elasticity have a position-dependent stiffness and cannot change that stiffness independently of the finger position, which is required in order to change applied forces on objects while keeping the same fingertip position, as implemented in the design of the Awiwi hand, part of the DLR Hand Arm System (Grebenstein et al., 2011). The variable impedance actuation (VSA) implemented in this robot (Friedl et al., 2011) allows the hand to be stiff without applying forces to an object; it achieves this by using a 2n tendon coupling and 20 DoF, driven by 40 motors located in the forearm (Friedl et al., 2015). Thus, the system is interesting for carrying out research on the optimal control of VSA and for in-hand manipulation, but is too complex for real industrial applications due to the high number of components and high cost. For this reason, we worked on the simplification and redesign of the Awiwi hand (Friedl et al., 2011) using new VSA finger concepts that are implemented in two grippers, WHISG (Wearable Hand to investigate Stiffness while Grasping) (Haas et al., 2018) and CLASH (Compliant Low-cost Antagonistic Servo Hand) (Friedl et al., 2018).

**Figure 1:**
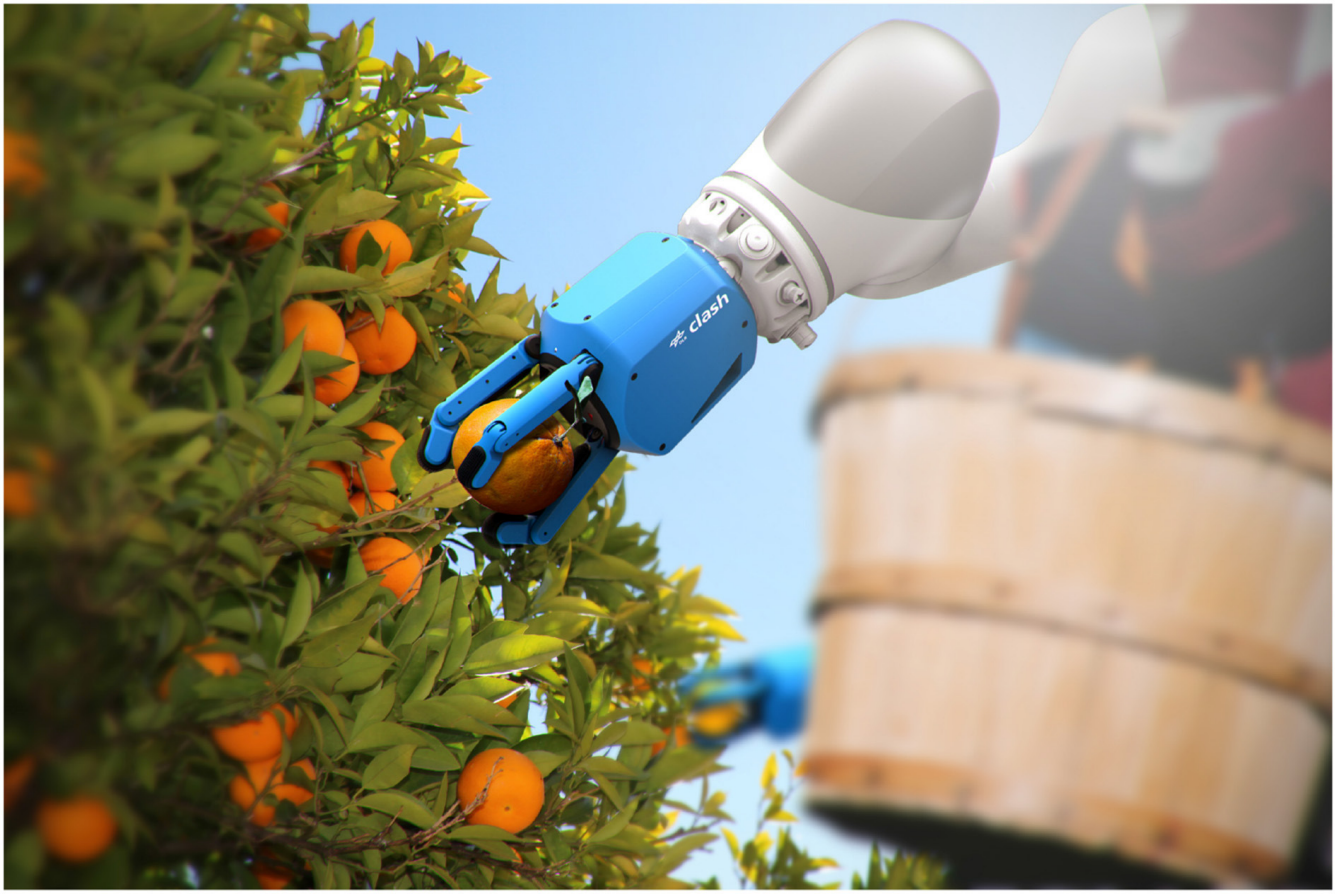
The DLR CLASH hand, a Compliant Low-cost Antagonistic Servo Hand built for robotic grasping of delicate groceries.

This paper is focused on the development and performance of the CLASH hand (Figure 1), a member of the new family of DLR hands based on the technology used in the Awiwi hand. The mechatronic design of the hand was initially presented in Friedl et al. (2018) and is extended here in section 2. The main contribution of this paper is the analysis of the potential application of the hand in real-world scenarios, as described in the following sections. This includes the implementation of a self-check and failure diagnosis system for the hand, which is presented in section 3. Section 4 theoretically analyzes the mechanical robustness of a stiff robot finger and the maximum Cartesian velocity required for finger safety and compares it with the expected performance for the CLASH hand fingers. Section 5 describes experiments that verify the robustness of the CLASH hand, and studies its grasp performance under failures. Finally, section 6 concludes the paper and discusses possible future work.

## 2. Design of the CLASH Hand

This section presents the main design concepts for the CLASH hand, which is based on the VSA design of the DLR Awiwi hand (Friedl et al., 2011). The number of DOFs, the fingertip forces and the joint velocity of the Awiwi hand are comparable to those of a human hand, but its weight and size are larger than a human forearm and hand, as all the actuators are located in the forearm, while the human hand is driven by a number of small muscles in the palm. Furthermore, the system is too expensive and complex to be used in real industrial cases. Based on the experience gained on the development and operation of the Awiwi hand, the following requirements were defined for the new DLR hand family:

1. The hand should have the potential to be attached to any robot, so the actuators should be located in the palm of the hand;
2. Modularity should be achieved, for instance using actuator boxes for the fingers;
3. The modular design should allow different finger configurations (e.g., 2- or 3-DoF, or lock-mechanisms) to be tested;
4. Fingers should be as strong as in the Awiwi hand, preferably using fewer actuators, so tendon coupling must be optimized;
5. The stiffness behavior of the fingers must be enhanced compared to the Awiwi hand;
6. The design should enable easy integration of different sensors into the hand (e.g., tactile sensors).

These requirements led to the design of new hand prototypes with three fingers, namely, the WHISG and CLASH hands. The hands provide research platforms for both hand-in-hand grasping (WHISG) and for doing grasp experiments with robots (CLASH). Both hands have three fingers: one opposable thumb plus two additional fingers, all based on the same design principles. Three fingers were chosen as an acceptable compromise between grasp capabilities and dexterity vs. mechanical and control complexity; in fact, it has been shown that, for anthropomorphic hands, three fingers is the minimum number required to obtain an acceptable level of dexterity (Saliba et al., 2013). The modularity at the finger level allows different possible kinematic arrangements for the CLASH hand, e.g., 2- or 4-finger hands are also possible. For the sake of clarity, hereafter, the term CLASH 3F will be used to specify results that are obtained for the 3-finger version of the CLASH hand.

### 2.1. Kinematic Optimization

**Figure 2:**
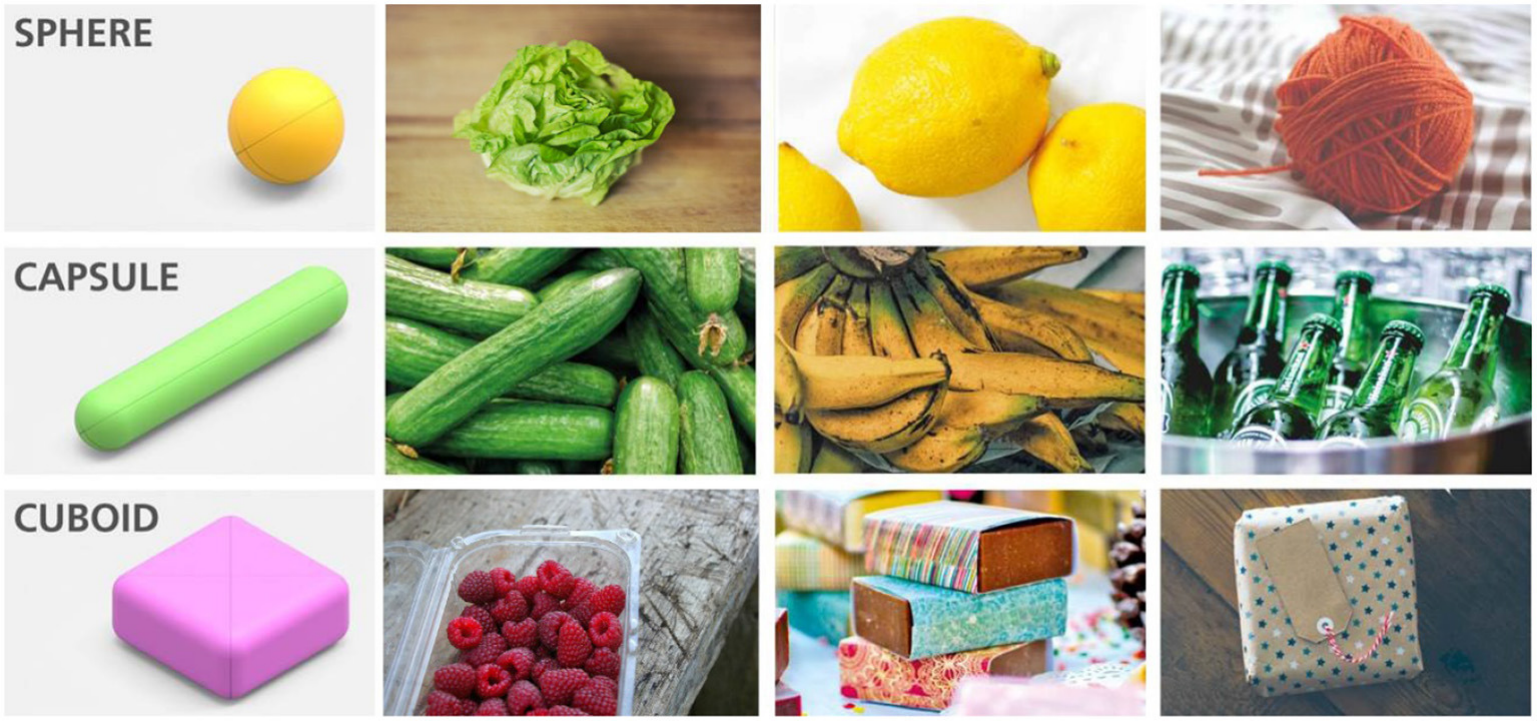
Different groceries (iceberg lettuce, cucumber, blueberry boxes) and their simplification for use in the kinematic optimization of the finger geometry in the CLASH 3F hand. The same primitive shapes can describe a large variety of objects. Adapted from Friedl et al. (2018) under the Creative Commons CCBY license.

The CLASH 3F hand has been designed through a geometrical optimization process considering the target objects coming from the SoMa project^1^, i.e., fruits and vegetables. The hand kinematics must be optimized for handling a prototypical set of groceries, selected as representative of the possible variations in shape for the use case, which include an iceberg lettuce (approximated by a sphere), a box of blueberries (approximated by a cuboid) and a cucumber (approximated by a capsule), as illustrated in Figure 2.

The initial design of the WHISG hand (Friedl et al., 2018) provided good behavior for changing stiffness with low loads; however, the variation in stiffness was rather limited when the loads were increased. Additionally, the fingers opposing the thumb (hereafter called the differential fingers) had a differential actuation mechanism that simultaneously changed the tendon force and the finger position, resulting in a deflection of the FAS (Flexible Antagonistic Springs). Moreover, we found that the differential coupling between the proximal and distal joints of the thumb also limits its variation in stiffness when pretensioning it. Due to these identified drawbacks of the highly underactuated fingers, we decided to improve the finger transmission system by adding extra servo motors and by reducing underactuation in order to obtain better stiffness variation.

**Figure 3:**
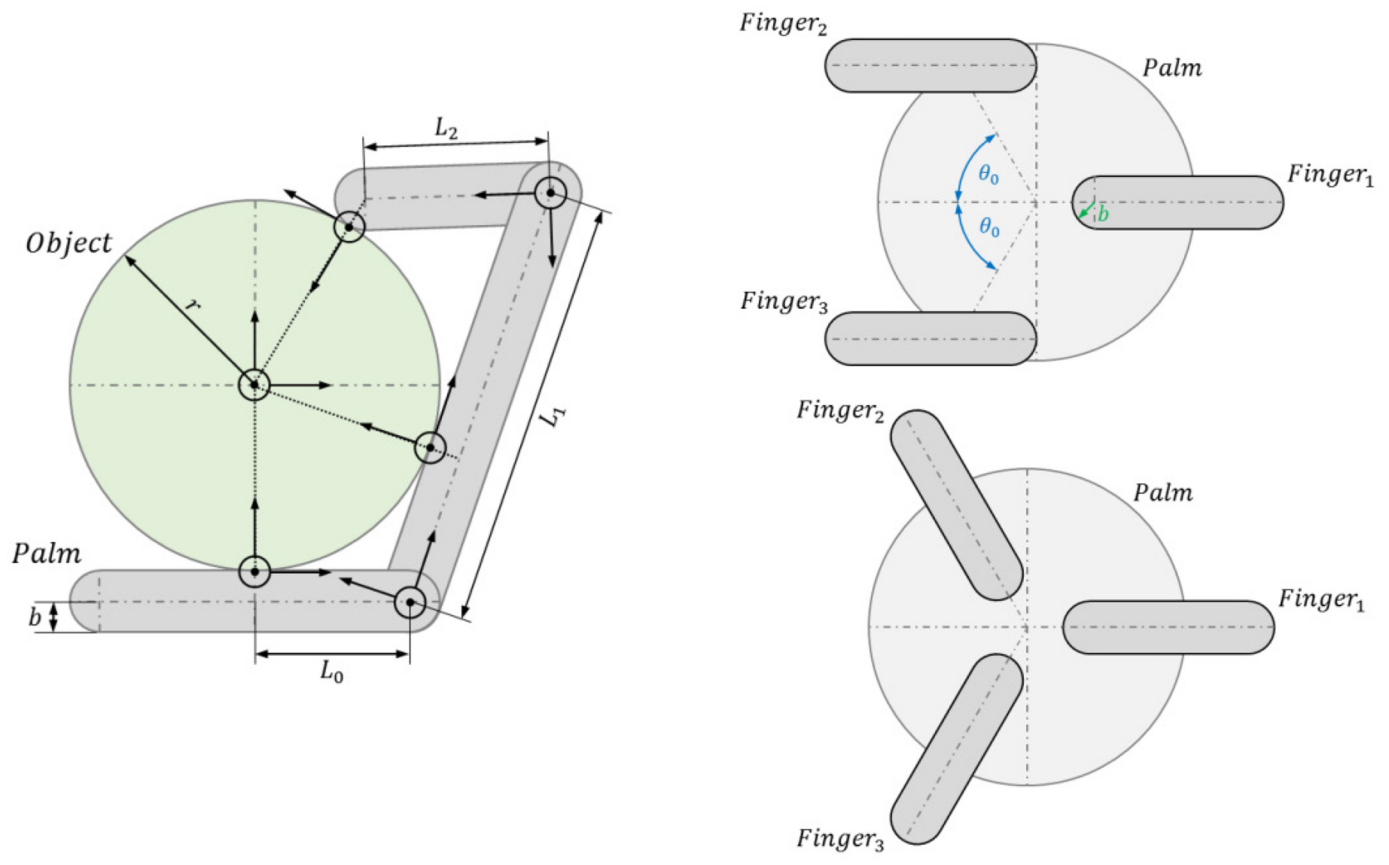
Parameters involved in the kinematic optimization process. **(Left)** Kinematic optimization of the length of palm and finger segments (in 2D). **(Right)** Optimization of the overall hand configuration and finger arrangement (in 3D) to define finger placement and orientation. Adapted from Friedl et al. (2018) under the Creative Commons CCBY license.

**Figure 4:**
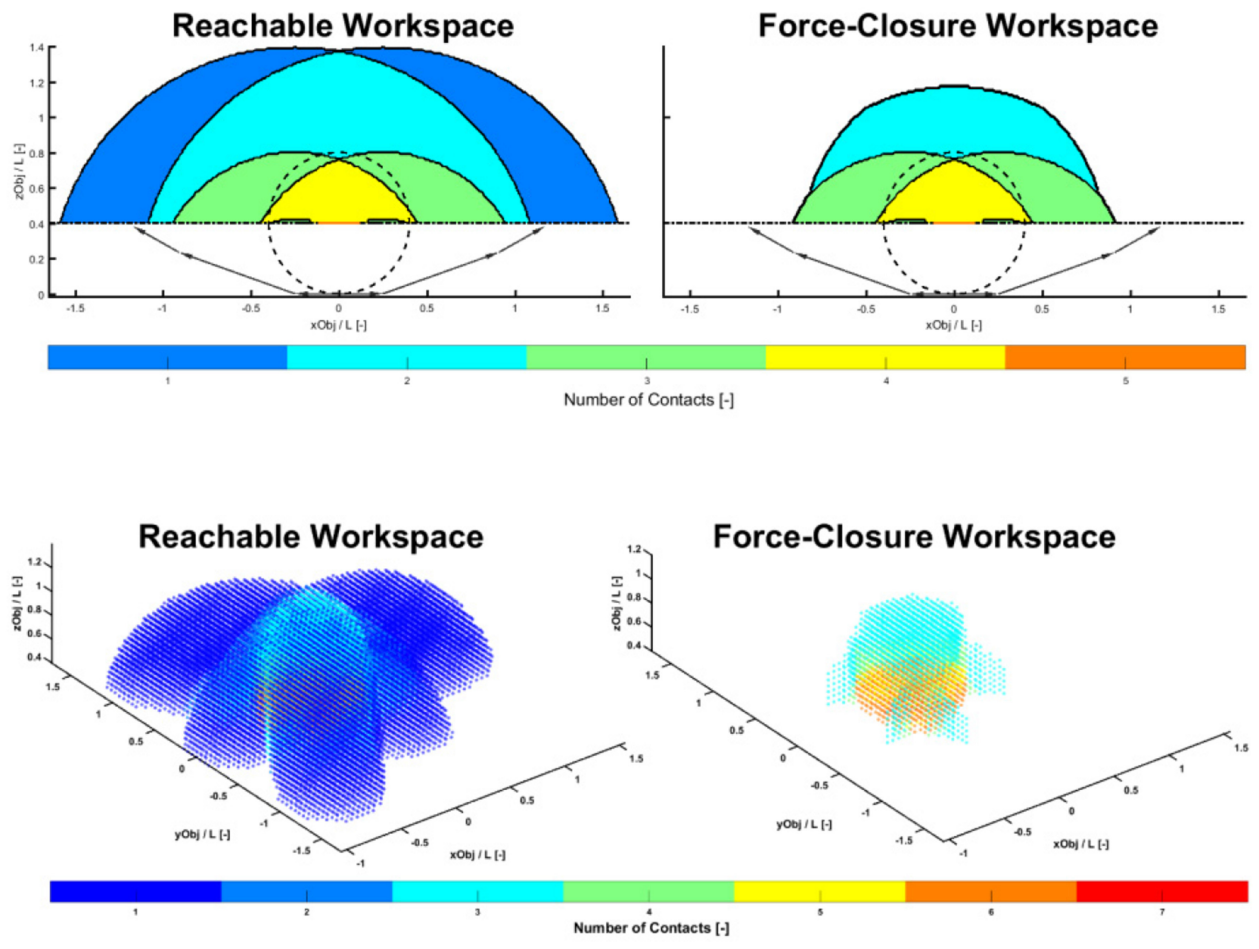
Reachable and force closure workspaces for the analysis in 2D **(Top)** and 3D **(Bottom)**. The color code indicates the number of contacts achieved on a spherical object placed at different locations with respect to the base of the hand.

We decided to investigate the kinematics with no proximal joints, so the new version of the fingers has two DoF for the fingers and three DoF for the thumb. The geometrical design of the CLASH 3F hand was carried out through a two-stage process. The first stage was geometrical analysis to obtain the segment lengths and the length of the palm for a planar hand (e.g., in 2D). Adimensional parameters were optimized for fixed relations of *r*/*L*, where *r* is the representative radius of the object (sphere, cuboid, capsule) and *L* the total length of the finger (Figure 3). The object is placed at different discrete positions with respect to the hand, and for each position, the hand is closed around the object to look for possible contact points. Different criteria can then be optimized, including the size of the reachable workspace, where the hand has at least two contacts with the object, and the size of the force closure workspace, where the contacts on the object lead to a force closure grasp (Figure 4). These criteria were analyzed for different values of *r*/*L*, *L*_0_/*L*, and *L*_1_/*L*, and the overall optimal geometry was selected as that which maximized both criteria. This process led to a palm baseline of *L*_0_ = 35 mm, total length of the fingers *L* = 100 mm, and link lengths of *L*_1_ = 70 mm and *L*_2_ = 30 mm when considering that the hand should grasp an object with a maximum radius of 70 mm. The second stage defined the overall placement and orientation of the fingers (in 3D) using the same process and optimization criteria as used in the previous stage. The results of the analysis led to a uniform distribution of fingers pointing toward the center of the palm.

### 2.2. Design of the Differential Fingers

**Figure 5:**
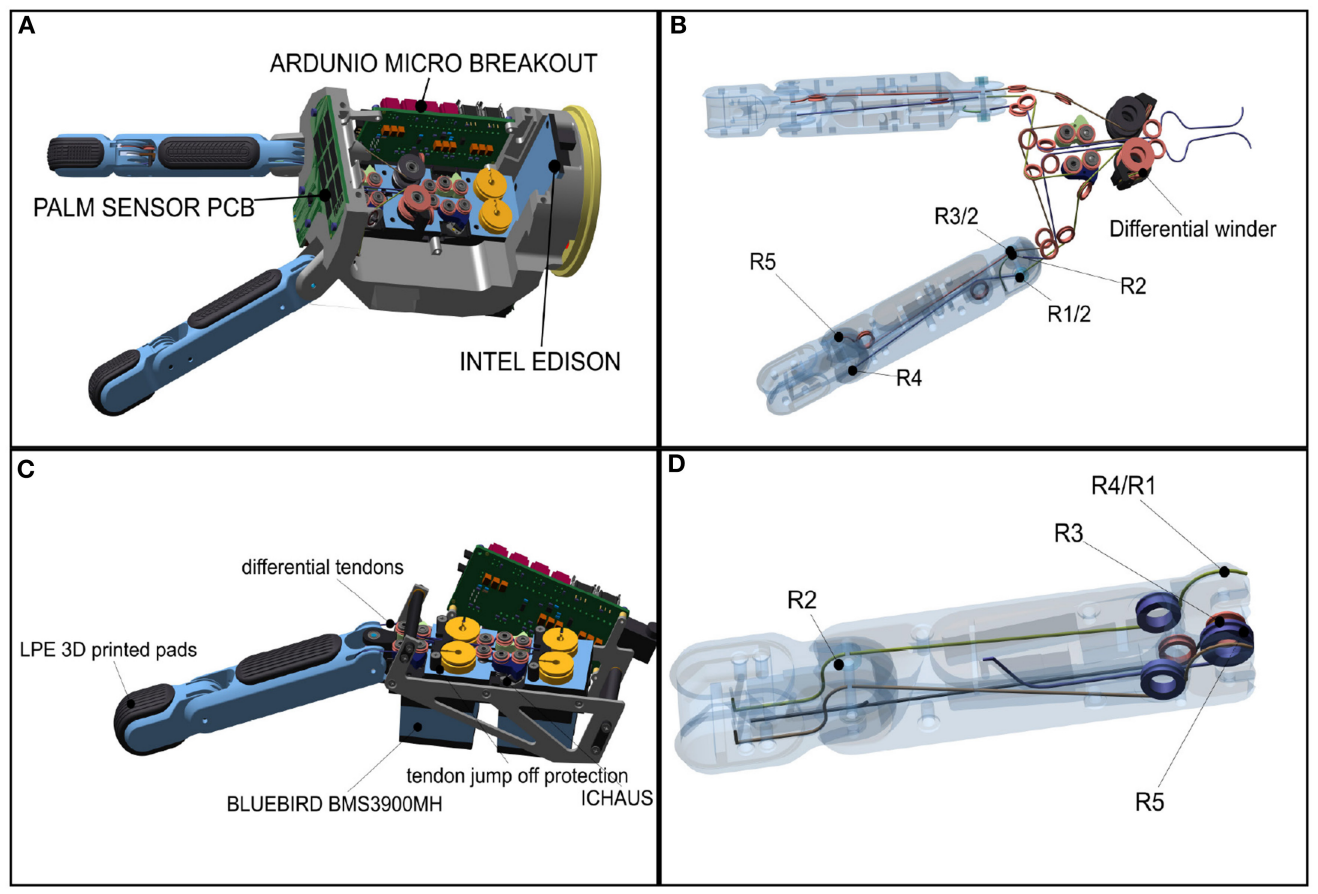
Mechatronic structure of the CLASH 3F hand: **(A)** differential finger module of CLASH, **(B)** tendon coupling for the differential fingers, **(C)** thumb module, **(D)** tendon coupling for the thumb. Adapted from Friedl et al. (2018) under the Creative Commons CCBY license.

To improve the modularity and capabilities of the hand, CLASH 3F uses the same servo module, with four motors for the thumb and for the differential fingers. The two extra servos of the module (compared to the module of the WHISG hand) are used to increase the fingertip force, the stiffness-variation capabilities, and the reachability of the finger. The metacarpal (MCP) joints of the secondary (differential) fingers can be actuated independently, but the distal joints of the two fingers are coupled, similar to the coupling observed between human pinky and ring fingers. In this way, the differential fingers have three active DoF. Using this new design, the maximum fingertip force is now 10 N for these fingers (four times the maximum fingertip force achieved previously with the WHISG hand). Figure 5 shows the tendon routing and the locations of the corresponding pulleys in the finger structure.

The following matrix describes the chosen tendon coupling for the 2 DoF differential fingers, i.e., it indicates how a given tendon (each individual column) affects a particular DoF (individual rows). The radii in the first row control the MCP joint of the left finger, those in the second row, the MCP joint of the right finger, and those in the third row, the coupled distal joints (for more details see Friedl et al., 2015):

(1) $$\begin{array}{l} R_{\text{differential fingers}}=\left(\begin{array}{cccc} -R_{1}/2 & R_{3}/2 & 0 & R_{2}\\ -R_{1}/2 & R_{3}/2 & R_{2} & 0\\ -R_{4} & R_{5} & 0 & 0 \end{array}\right) \end{array}$$

### 2.3. Thumb Design

Due to the increased fingertip force of the differential fingers, the thumb was redesigned to increase its force capabilities by a factor of two to effectively oppose and resist the forces created by the differential fingers. Inspired by human tendon routing, the two flexor tendons for the MCP joint now end at the proximal joint. In a human, the strongest finger tendon is the profundus tendon, which ends at the distal bone and can generate torque on all prior joints. This approach is followed by most tendon-driven underactuated hands using only one tendon, while in the CLASH hand we use two tendons for the same purpose (Figure 5). The differential coupling between the joints improves its ability to deal with environmental constraints, in particular allowing sliding of the fingertip over a surface to reach flat objects, as it reduces the control requirements due to the finger self-adaptation. In other words, this allows an open-loop control of the sliding motion if the extensors are controlled by a soft admittance control. On the other hand, this routing leads to a very strong distal joint (PIP) and a weak MCP joint. To solve this situation, the proximal extensor tendon is used as a flexor in the MCP joint, as described by the tendon-coupling matrix:

(2) $$\begin{array}{l} R_{\text{CLASH thumb}}=\left(\begin{array}{cccc} R_{4} & -R_{4} & 0 & 0\\ R_{1} & R_{1} & -R_{5} & R_{3}\\ R_{2} & R_{2} & 0 & -R_{2} \end{array}\right) \end{array}$$

**Figure 6:**
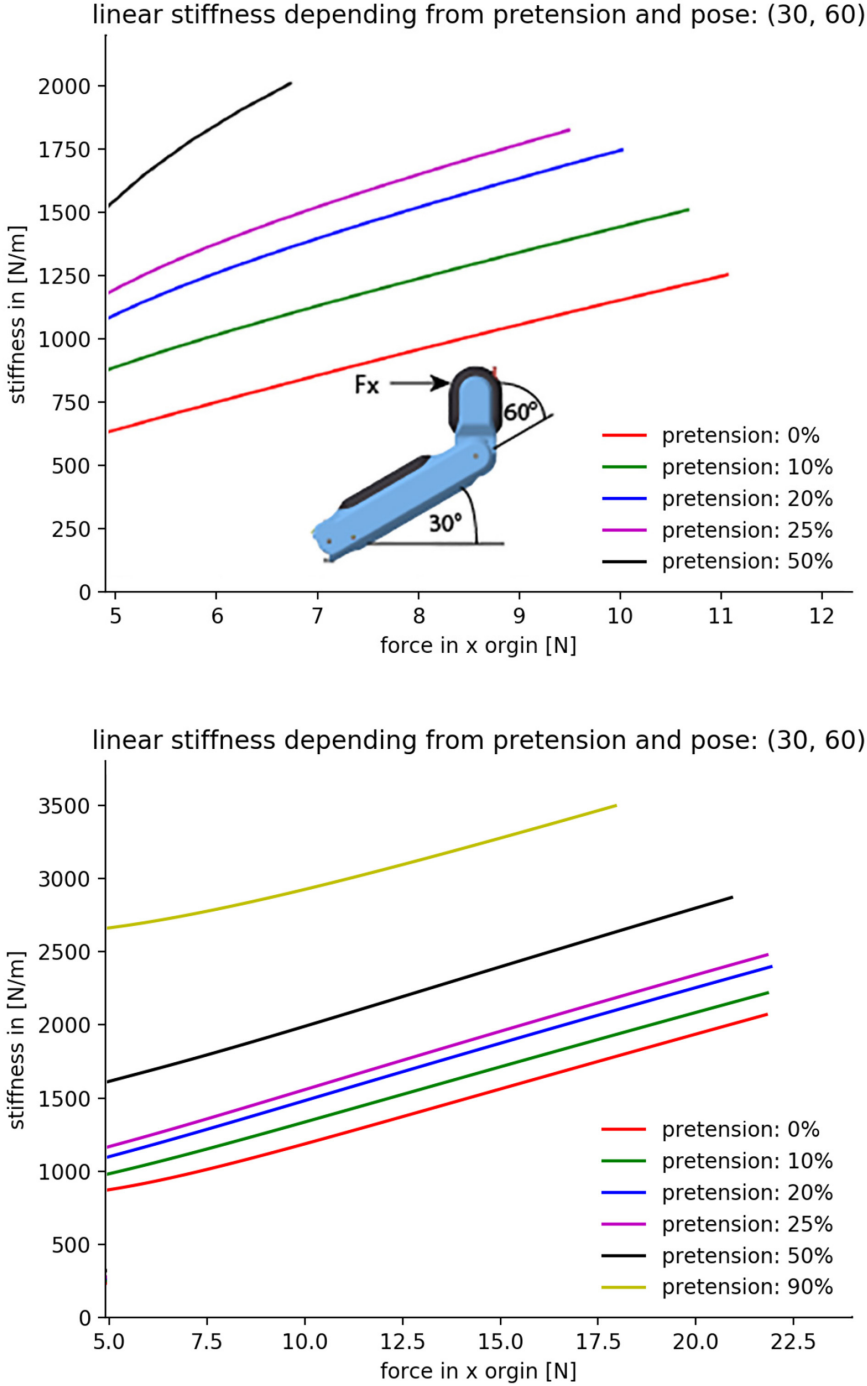
Experimental evaluation of force-stiffness profiles for the thumb in the WHISG **(Top)** and CLASH **(Bottom)** hands. Start position: base 30°, distal 60° (depicted in the sketch at the top). The horizontal axis corresponds to the force in the x direction applied at the fingertip; 5N are generated by the contribution of all springs in the finger. The finger is deflected along the x direction until one tendon reaches the maximum tension of 65N; force in y direction is zero. Repeated by increasing pretension in 10 % increments. Adapted from Friedl et al. (2018) under the Creative Commons CCBY license.

**Figure 7:**
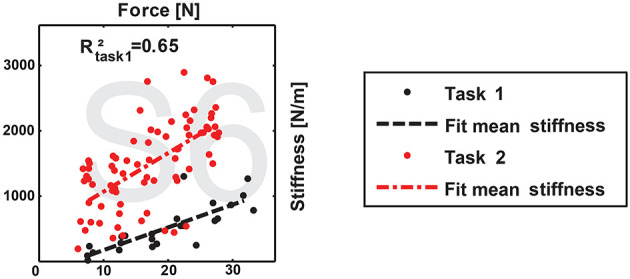
Exemplary diagram of human grip stiffness. Adapted from Höppner et al. (2017) under the Creative Commons CCBY license.

In this new tendon routing, two tendons work in the base and the distal joint as flexors, and the extensor of the distal joint acts also as a flexor in the base. This design allows a maximum fingertip force at the thumb of 20 N and considerably increases the range of stiffness variation under load in comparison to the WHISG hand, as shown in Figure 6. The result looks qualitatively similar to the force-stiffness diagram measured for the human pinch grip (Figure 7).

**Figure 8:**
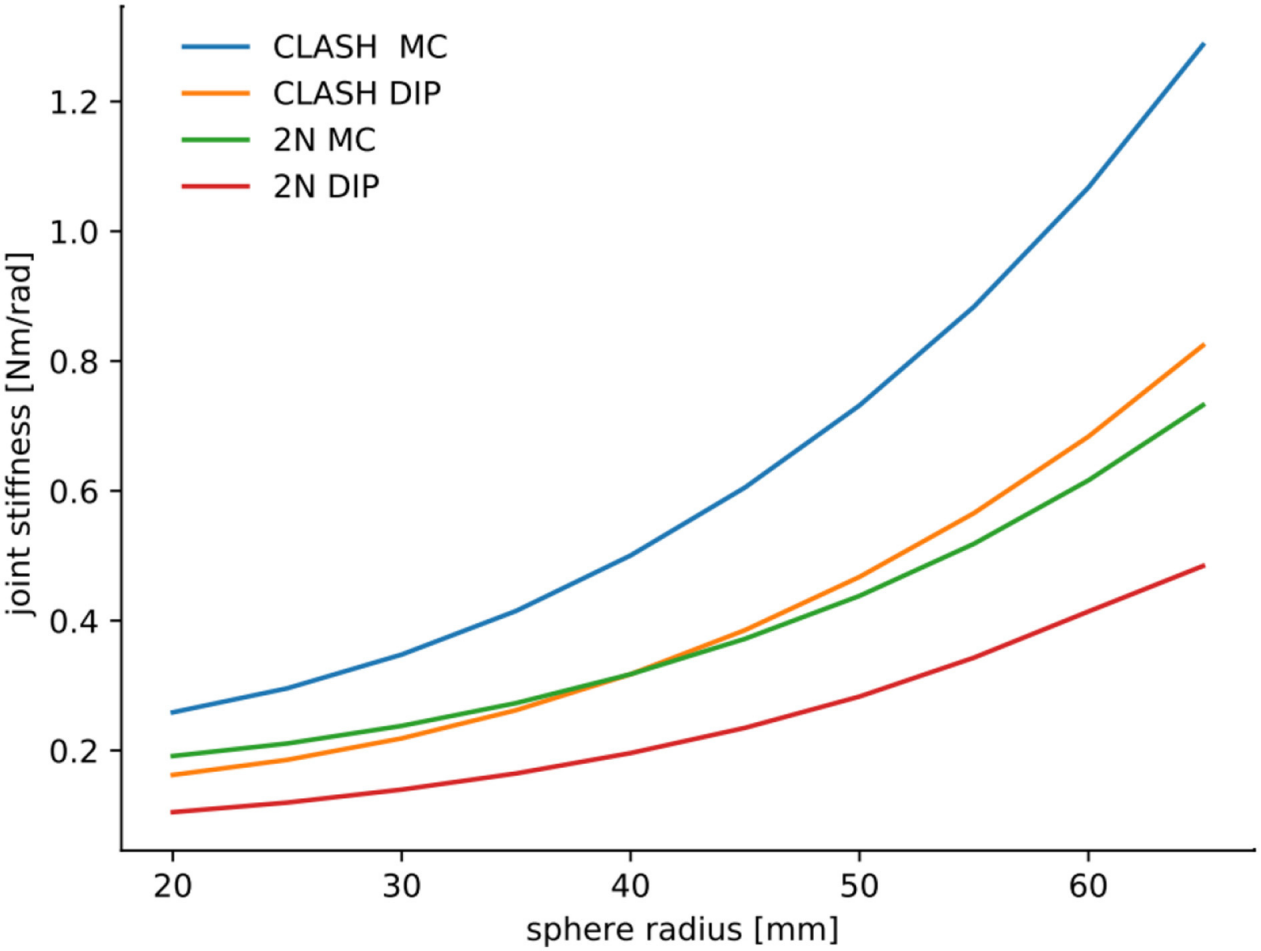
Comparison of object-dependent stiffness between the tendon routing for the CLASH and a 2n design. Adapted from Friedl et al. (2018) under the Creative Commons CCBY license.

To analyze the hand behavior when grasping an object, a simple planner was used to compute the grasp forces required to grab a sphere from the top. The material of the sphere was assumed to have a density equal to water, similar to most fruits and vegetables, e.g., peaches, apples, cucumbers, and mangoes can float—potatoes and tomatoes sink. This leads to the computation of an object-dependent stiffness, as shown in Figure 8. The figure shows that the stiffness for the CLASH hand increases faster than, for example, the 2n coupling of the 3-DoF fingers, which are used in the 2-finger hand of the DLR Hand Arm System. The result for the WHISG hand is not presented, but it is quite similar to a 2n design. The faster increase of stiffness leads to a more stable grasp against weight variations of the gripped object.

### 2.4. Electronics, Sensor, and Software Concept

**Figure 9:**
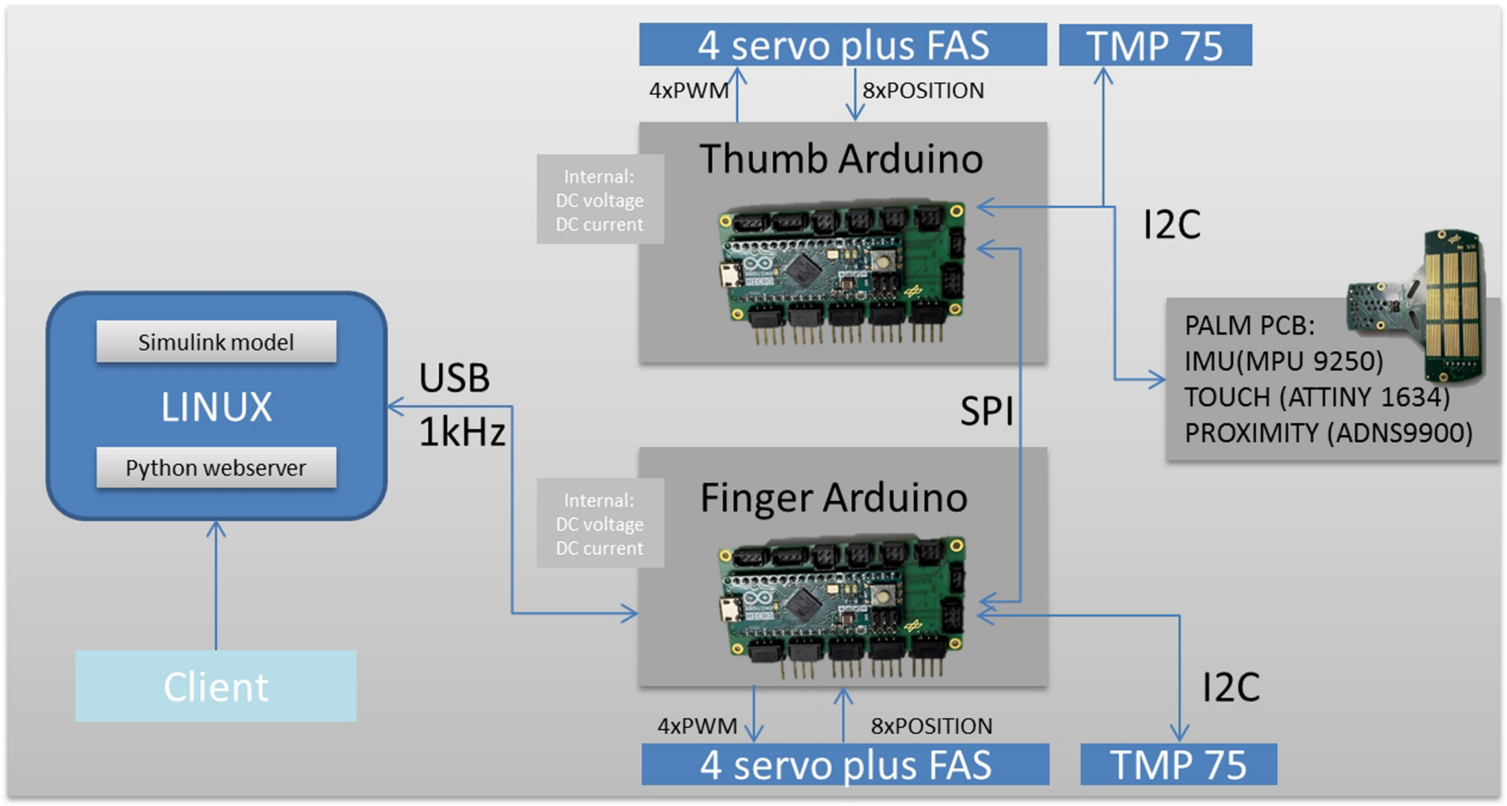
Electronic design of the CLASH 3F hand.

To reduce the cost and complexity of the system, the CLASH 3F hand uses Arduinos to control the servos and to collect sensor data (Figure 9). The two Arduino Micros (Atmel Mega32U4) can control up to five servos with their timers. The servos used in this hand are Bluebird BMS-3900 MH. The potentiometer values of the servos are fed back to the Arduinos to calculate all finger positions. The angles of the variable stiffness levers are measured by an analog Hall sensor (ICHaus MP), which works well with the magnet from the Awiwi FAS. The deflection of each lever is introduced into the FAS model to obtain the tendon force. The tendon force is then used, together with the coupling matrix, to estimate the joint torques used in the admittance control of the hand.

Both Arduinos communicate with each other via their SPI interface. The SPI master has a USB connection to a Linux computer, which in the case of the portable version of the CLASH 3F is an Intel Edison board. This board runs the executable code generated by a Simulink model, a driver process handling the USB communication, and a web server providing access to hand parameters. Inter-process communication is provided by the DLR Links_and_Nodes communication middleware. The USB communication uses synchronous transfers with a payload length of 32 bytes in both directions at a rate of 1 kHz.

The palm is equipped with a sensor board that has an IMU to acquire the orientation of the hand, a proximity sensor to detect objects before contact, and a small microcontroller (Atmel Tiny1634) to collect data from palm and fingertip tactile sensors. The tactile sensor is based on the piezoresistive effect of the 3M Velostat foil or ESD foam (Koiva et al., 2013). The palm has a 3 × 3 sensor area, and all fingers can be equipped with tactile fingertip sensors with 3 × 3 tactels on each sensor. The sensor board is connected via I2C to the thumb Arduino board. In normal operation, the three I2C sensors are read at a rate of 166 Hz. If fast tactile information is required, the point of interest can be switched so that touch information can potentially be updated at 500 Hz. Additional sensors can still be connected: the SPI master has a free I2C and UART port that could, for example, be used to integrate the SPAKFUN Robotic Finger Sensor (Patel and Correll, 2016). Two TMP75 sensors measure the temperature of the servos, and the internal states of DC link voltage and current are collected.

### 2.5. Robustness in Comparison to Other DLR Hands

Table 1 presents a comparison of different finger features among a number of DLR hands. The comparison is performed at the finger level due to the modularity of the CLASH hand, i.e., it would be possible to build a hand with a similar kinematic structure to the DLR Hand II or the DLR/HIT Hand using the thumb module of CLASH 3F. In terms of robustness it is clear that the hands with VSA (Awiwi, WHISG, and CLASH) are much more robust than the stiff hands (DLR Hand II, HIT Hand II, and DEXHAND). This is also reflected in the daily usage of the hands in the labs—a planning error that results in a collision can lead to damage in the stiff hands if the human does not react fast enough to stop the collision. This is not generally a problem for the VSA hands, which can, in principle, resist unintended collisions. A tendon breakage due to an impact is very unusual; a collision might lead to one of the tendons jumping off the pulleys or guides, which could later lead to a sliced tendon, mostly in the fingers. This problem is prevented in the Awiwi hand at the control level because a mechanical solution was not implemented. The tendon pretension controller tries to hold a minimum pretension of 12 N. As a result, the hand loses about 40% of the passive deflection, and the hand is mostly used in a medium-stiff configuration. This behavior led to the development of a mechanical jump-off guiding system in CLASH, where the whole spring deflection can be used to increase the robustness and softness of the hand. Furthermore, the concept of end stops for zeroing the hand is not needed anymore in all joints, because the RC-servo provides an absolute position reading, which was not available with the Awiwi actuators. The end stops reduce the possible range of motion, reducing the potential damage if a crash happens at the palmar side. The Awiwi hand has joints that can dislocate mechanically, but the preloaded tendons prevent the joint dislocation. The CLASH fingers also use the ability to dislocate, which might happen in the case of hits from a lateral direction. Indeed, these fingers do not have a DOF for lateral motion, so the joint has to dislocate if such a hit occurs. The dislocation is supported by the compliance of the joint tendons; the finger can be relocated easily after such an event. Therefore, the overall robustness of CLASH is better than that of the Awiwi hand, even though the Awiwi hand uses steel instead of nylon tendons.

**Table 1:** Finger properties for different DLR hands.

**Hand**	**Fingertip force (N)**	**Module weight (g)**	**Robustness**	**Approximate cost (Euro)**
Hand II	25	408	o	2, 000
HIT Hand II	8	250	o	2, 000
DEXHAND	25	440	o	3, 000
Awiwi II^a^	20	700	+	16, 000
WHISG	10	200	+	300
CLASH 3F	20	200	++	300

a *Awiwi hand with steel tendons (Friedl et al., 2015)*.

## 3. Self-checking and Diagnostic of Failures

**Figure 10:**
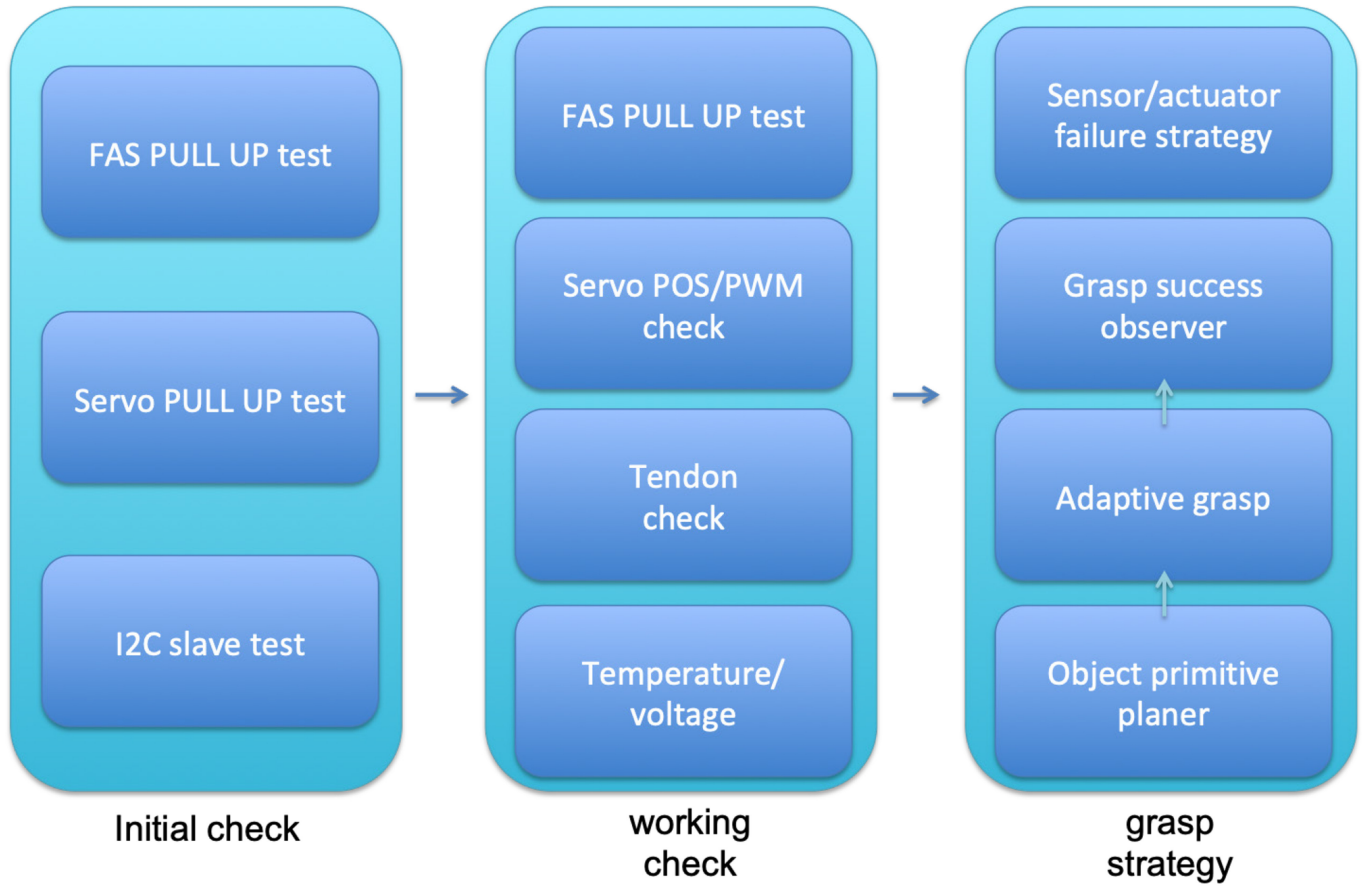
Self-checking systems of CLASH hand during startup, working conditions, and grasp execution.

For industrial applications, self-checking of the hand is important to enable the detection of defective hardware as fast as possible to keep the production line running. In a research lab environment, the task would be stopped and the manipulator replaced, which is not acceptable in a real-world application. If the hardware problem is identified, the system can choose a compensating strategy to use the manipulator with the current limitations, and the task can still be performed while the service staff is on its way to repair it. Due to the hand design based on differentially-coupled joints, for instance, a failure in one of the flexor motors or tendons can still be compensated by the other flexor. The combination of self-checking and differentially-coupled joints helps to maintain a certain level of grasp performance even though failures are present. The self-checking of CLASH is triggered in an initial diagnostic check after power-up, and systems are checked during normal working conditions, as presented in Figure 10.

An initial diagnostic is done by the firmware of the two Arduino micros. The main function is to check the cable connections between sensors and actuators. Pulled-up resistors, for instance, show disconnected tendon force sensors and motors (detected with the FAS and Servo pull up tests in Figure 10). The FAS pull up test can be performed also during normal working conditions, but not the servo pull-up tests, as the motors cannot be checked in working conditions by the resistors because they disturb the position controller. However, the number of I2C slaves is verified, to check if all of them are still connected; if not, then an initialization error is reported, and eventually a compensating action could be implemented so that the hand can work until it is serviced. If a failure state arises during the initial check, the system will perform a full system check and then compare and report the failures.

During operation, the system can also check whether one of the tendons is broken by increasing the passive stiffness of the system and comparing the nominal and real spring deflection. Also, the servos can be checked by verifying that the commanded and real positions match within an allowed threshold (Servo POS/PWM check in Figure 10). Another parameter that is monitored during operation is the temperature of the RC servo. Off-the-shelf servos often have no internal temperature sensor or management due to the low cost of the part; therefore, the hand is equipped with three temperature sensors, two between the main flexor servos of thumb and fingers, and one in the palm, to check the overall temperature. If one of the sensors between the flexors shows overheating above 55°C, the admittance control is activated and a warning is sent out. The admittance control then reduces the torques and allows the motors to cool down. If the temperature rises above 60°C the hand goes back to zero position and zero stiffness with a still-operational admittance control. If the temperature goes down again below 50°C, the warning and the admittance control are deactivated.

To support the grasping of new objects, we developed a simple grasp planner that can calculate for objects with simple forms the necessary finger positions, torques, and tendon forces. This is especially important for real applications with sensitive objects in order to get a good starting pose for a successful grasp. Also, these values can be used by a grasp observer, which provides an initial estimation of the grasp quality before lifting the object, and shows whether an object is lost during the grasp attempt. Based on the sensor data from our tendon force sensor and the proximity sensor, the grasp observer can help with learning better strategies for grasping. The CLASH hand was initially presented at the Automatica trade fair in 2018, where we used a vision-based grasp success observer, but the quality of this estimation was not satisfactory. Additional sensor modalities were incorporated into the observer to get an operational reliability above 99%. For top grasps, for instance, the proximity sensor can detect objects slipping out of the hand, and so a reflex was included in the hand control to cope with this situation. The detection of slippage is even more robust when the information coming from the torque observer is also considered.

## 4. Analysis of Finger Robustness

In the absence of high quality sensor feedback, a hand requires passive compliance to be robust in real applications (Friedl et al., 2018; Haas et al., 2018). This section analyzes if higher quality sensor signals really help even in the case of stiff hands. For this purpose, we analyze in more detail the transfer phase of a manipulation action, i.e., moving the object from the picking to the placing location. During this transfer phase the robot should be as fast as possible to be time efficient. If the fingertip contacts the environment, e.g., a container, due to planning or execution errors, a stiff hand has to react to reduce the collision torque and withstand the impact. To illustrate this point we take the DLR Dexhand equipped with the same steel tendons used in the Awiwi hand. This concept of tendon-driven fingers can be found also in DLR Hand II and DLR 5-finger hand. The calculation that follows tries to find a safe velocity for moving the robot hand such that it would allow to withstand a possible collision without damaging the finger motors. The parameters used in the calculation are presented in Table 2.

**Table 2:** Parameters for calculation of finger robustness.

**Parameter**	**Value**	**Unit**
Finger length, *L*	100	mm
Contact point, *c*	20	mm
Max tendon force, *f_*max*_*	150	N
Motor inertia, *I*	7*E*−9	*kg*/*m*^2^
Max motor velocity, *v_*m*_*	12,000	rev/s
Command delay, *t*_*delay*_	4	ms

**Figure 11:**
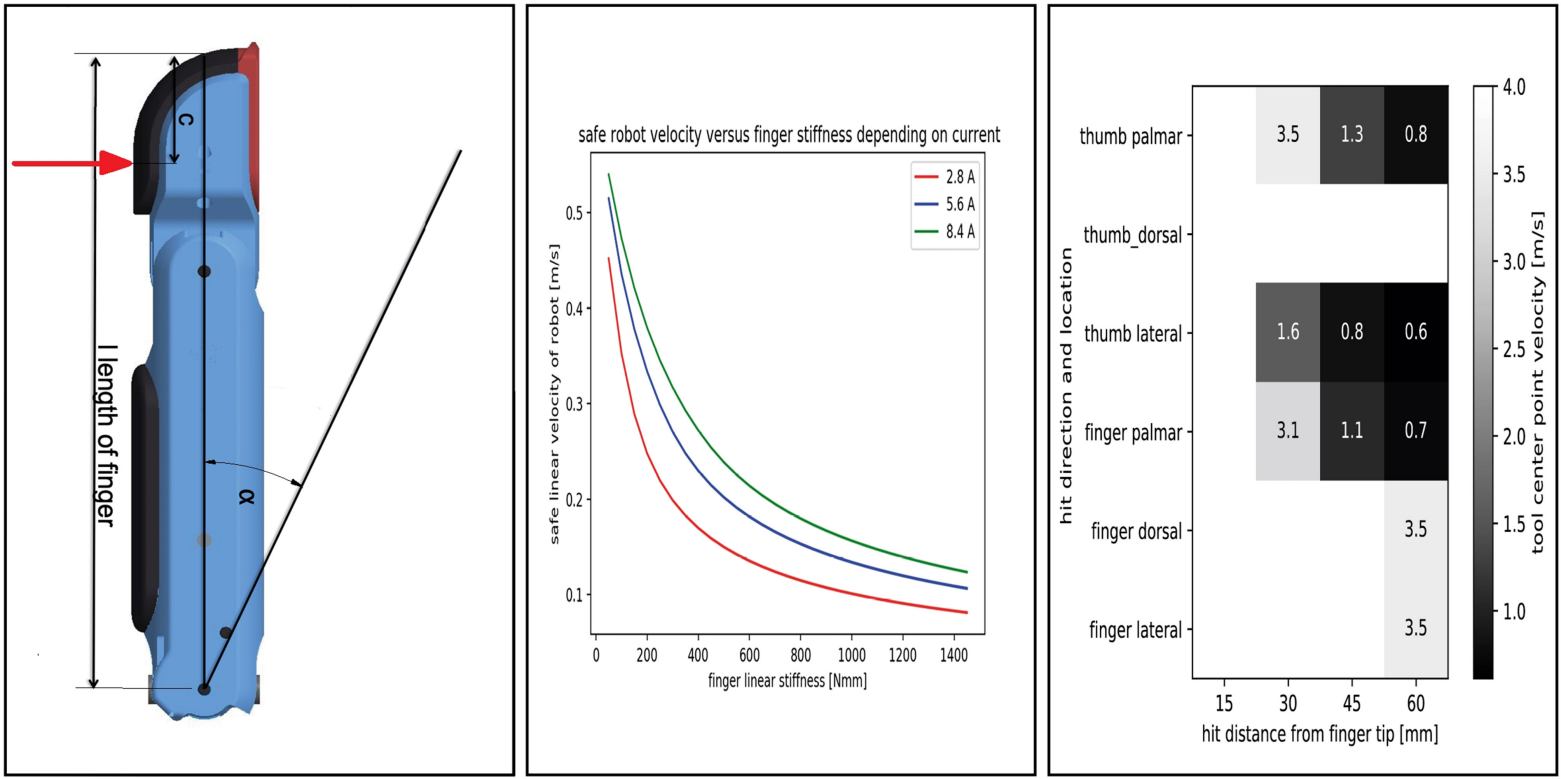
Analysis of finger robustness. **(Left)** Parameters used in the analysis. **(Center)** Safe robot velocity for a stiff hand and fingertip. **(Right)** Safe robot velocity for CLASH.

First we compute the angle α that the finger has to move, if the contact happens 20 mm away from the fingertip

(3) $$\begin{array}{l} \alpha =arccos\left(\left(L-c\right)/L\right) \end{array}$$

where *L* is the total length of the finger and *c* is the distance to the applied force, measured from the fingertip, as depicted in Figure 11. Assuming constant angular acceleration for the servo motors, one can compute the time required to reach the maximum angular velocity, *t*_*acc*_, and the time that the motor should run at maximum speed, *t*_*vel*_, to cover the angular distance α.

The next important parameter is the joint stiffness, which defines the maximal deflection of the joint that does not damage the tendon. For a simplified computation, we neglect the structural and gear stiffness and consider only the tendon stiffness, as it normally has the smallest value in the system. From the measurements with the Awiwi hand, a tendon with 7 × 19 composition has a maximum elastic module of 150,000 N/mm^2^. Thereby, the linear stiffness can be calculated as follows:

(4) $$\begin{array}{l} k=EA/L_{t} \end{array}$$

where *L*_*t*_ is the tendon length and A is the cross-sectional area of the tendon. With the known stiffness and the sample delay *t*_*delay*_, we can now calculate the maximum safe linear velocity for the hand as

(5) $$\begin{array}{l} v=\frac{f_{max}*\left(L-c\right)}{k*R_{1}*\left(t_{acc}+t_{delay}+t_{vel}\right)} \end{array}$$

Whether this velocity is achievable or not depends on the maximum current and rotational velocity of the motor.

For a tendon length of 150 mm, we get an equivalent linear stiffness of around 770 N/mm. Figure 11 presents the results of safe velocities required to protect the hand. For the transfer phase of a manipulation task, the results show a great limitation in the allowable speed, which would increase the time lost in this phase.

To calculate the safe robot velocities for CLASH, we can look first for the passive protection and then on the passive plus active reflex protection. The active reflex is triggered if the velocity of the spring deflection rises above a certain threshold. If this happens, the fingers drive as fast as possible away from the collision. For impacts at velocities lower than 0.7 m/s the finger can drive away from the impact at any distance from the fingertip. If we compare it with the stiff hand impact distance from Figure 11, the VSA fingers allow more than eight times higher end effector velocities compared to the stiff hand design. Note that this is possible even though the drive-away velocities are based on the low cost RC-servos use in the CLASH hand, which have 1/10 of the motor power used in DEXHAND.

## 5. Experimental Verification of the Hand Robustness

**Figure 12:**
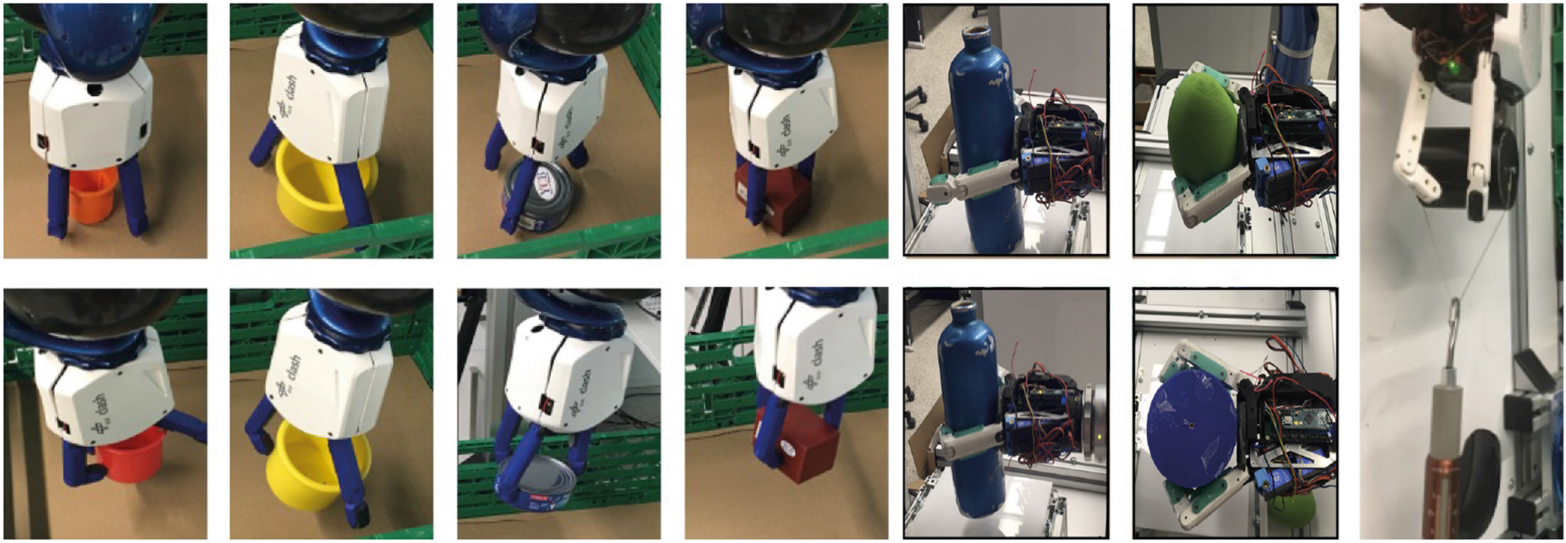
Grasping different household objects with pinch or power grasps. The rightmost picture shows a pull-out test for a cylinder with diameter 60 mm, resulting in a pullout force of 28 N. Pinch grasps and pull-out test pictures are adapted from Friedl et al. (2018) under the Creative Commons CCBY license.

**Figure 13:**
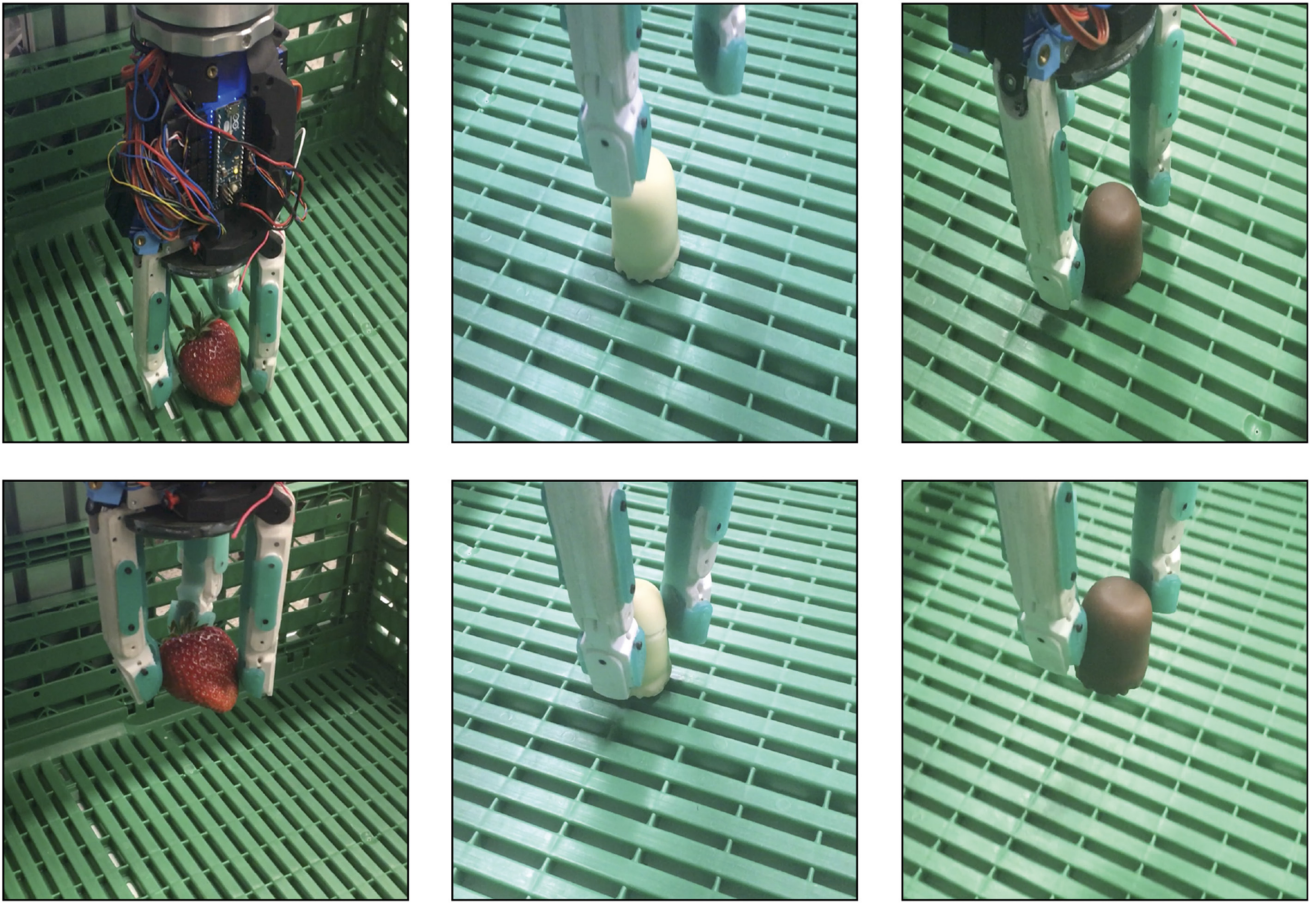
**(Left)** Grasping a strawberry with a contact fingertip force threshold of 0.3 N. **(Middle)** Grasping a whippet cookie with a fingertip force of 1 N, which damages the cookie. **(Right)** Successful grasp of a whippet cookie with a fingertip force threshold of 0.2 N.

The CLASH hand is capable of handling a large variety of objects, as shown in Figure 12, using both power and fingertip grasps. In previously reported experiments, for instance, at the Automatica 2018 trade fair^2^, the hand achieved a success rate of over 95% in pick and place actions. Furthermore, the hand can also grasp delicate objects like whippet cookies or strawberries, as shown in Figure 13.

This experimental section is focused on the experimental verification of the robustness of the CLASH hand. The first part looks into the robustness of the system, while the second part is focused on the grasp performance when a hardware failure occurs, including, for instance, a dead motor, sensor error, or broken tendon.

### 5.1. Robustness Tests

**Figure 14:**
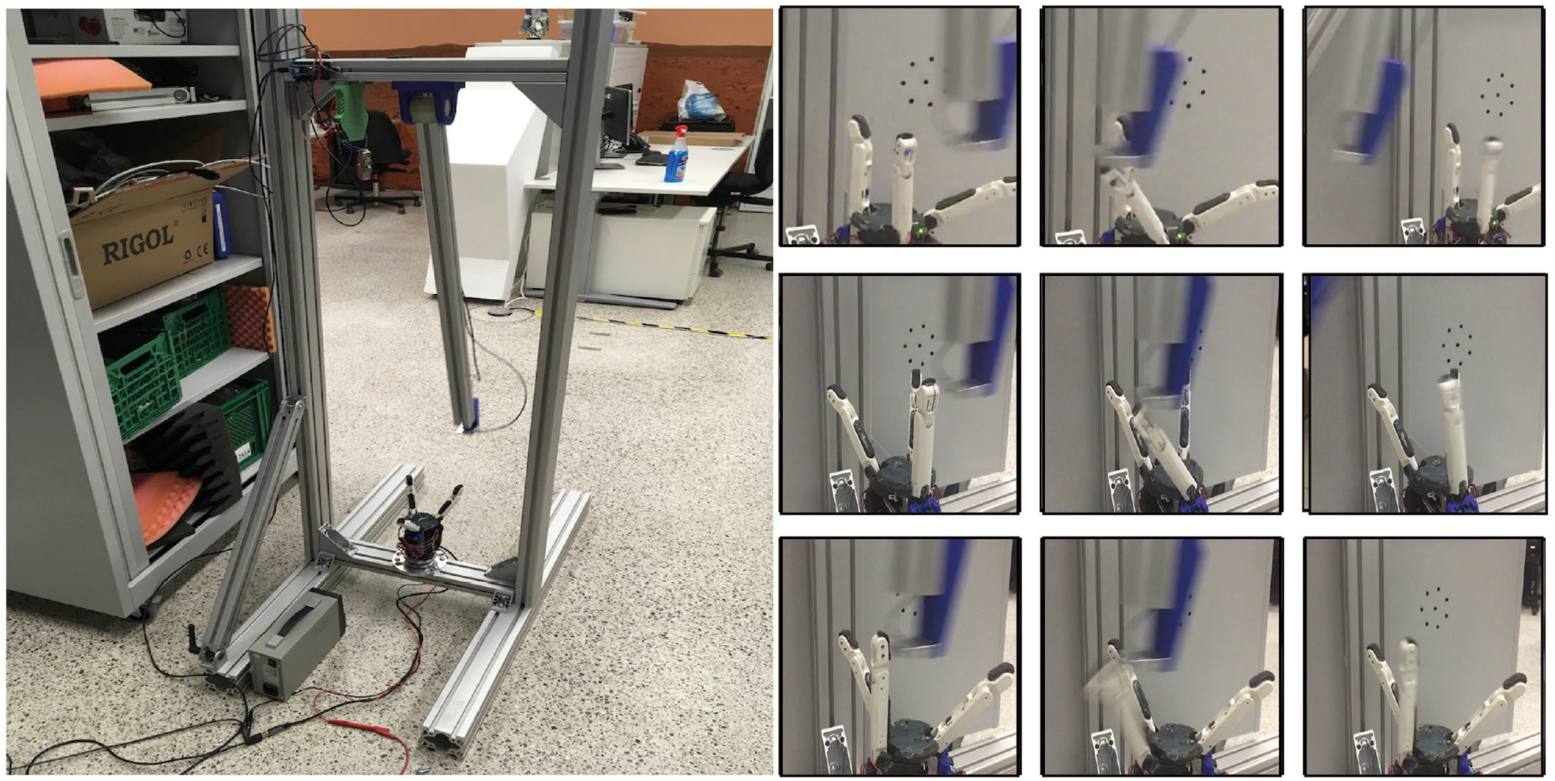
Impact experiments. **(Left)** Pendulum with 50 g accelerometer and 2 kN force sensor. **(Right)** Top row: lateral impact on one finger; middle row: lateral impact with dislocation of thumb; bottom row: palmar impact on one finger.

For the robustness tests, we use an impact pendulum, as presented in Figure 14. The experiments can be divided into passive robustness and active reaction. From the theoretical hand resilience map (section 4), we know the robot velocities that the hand can withstand when an unintended collision happens. The velocities were reproduced with the impact pendulum, as illustrated in Figure 14, and the hand effectively survived the predicted velocities.

**Figure 15:**
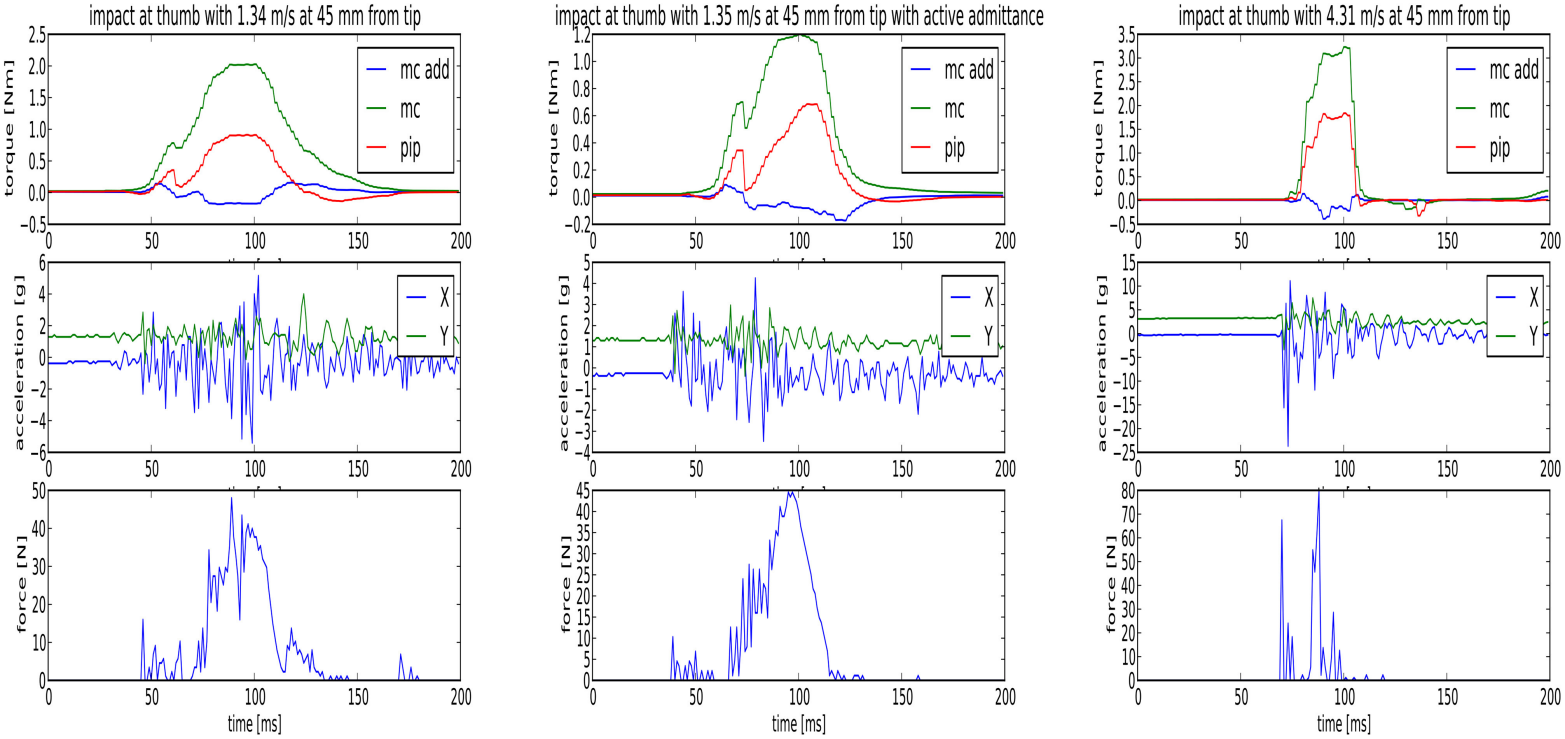
Active reaction. **(Left)** Reaction force on hand and pendulum after impact. **(Center)** Reaction force on hand and pendulum after impact with active admittance control in the fingers. **(Right)** Reaction force on hand and pendulum after impact with drive-away strategy.

A more interesting aspect is the effect of an active reaction, i.e., if the hand survives higher impact velocities for hits at the base phalanges of the thumb or differential fingers. For this experiment, we hit the thumb at a distance of 45 mm from the tip. Thus, the hit occurred at the proximal phalanges (the length of the distal phalanges is 30 mm). The results of the impact test can be seen in Figure 15. The pendulum energy for an impact at 1.34 m/s is too low to destroy one of the tendons, and so the finger can absorb the impact energy. The torques reach the maximum possible active motor torque of 0.47 Nm. The force sensor in the pendulum shows a first peak when the striker hits the thumb and then the highest pike when the finger compliance is fully used to absorb the impact. Now, the effects of the impact can be minimized by using the admittance control of the thumb. The admittance controller uses the torques measured based on the calculated forces from the spring sensors. The results are presented in Figure 15: the resulting torques are reduced by 40% and the reaction acceleration by more than 80% compared to the previous case.

In contrast to variable impedance actuators with fixed bearings, as used, for example, in the joints of the DLR Hand Arm System, the fingers can dislocate in the CLASH hand. Thus, it is possible to reduce the tendon preload to produce slack, which allows the fingers to dislocate more easily and therefore withstand higher impact velocities. For the last test, we implemented a thumb deflection velocity observer, which can trigger motion of the motors that leads to slack in all tendons. Under this condition, the finger withstands an impact at more than 4.3 m/s without major damage; the only effect is that the proximal silicon pad is kicked out of its clamping position. As shown in Figure 15, the resulting tendon forces are much higher than those that the motor can generate (considering that the motors together can generate up to 2 Nm), but they are still below the break load of the tendons.

### 5.2. Grasp Performance Under Failures

**Figure 16:**
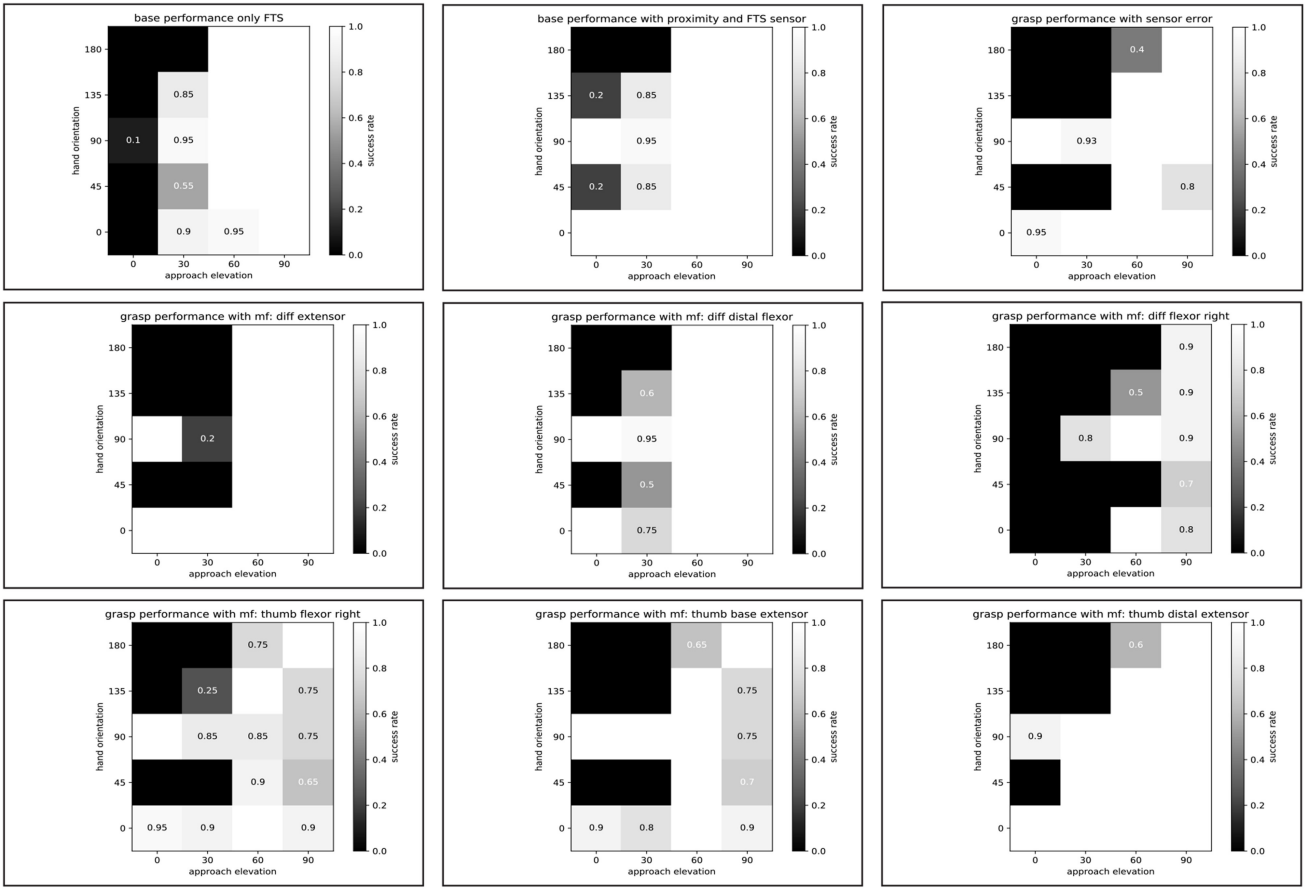
Grasp performance under failures. **(Top)** Ground-truth grasp performance with FTS sensor, with FTS and proximity sensor, and with sensor error. **(Center, Bottom)** Grasp performance with different motor failures (mf).

To test the performance of the hand, we used a benchmarking test developed for the specific use case of handling fruits and vegetables (Sotiropoulos et al., 2018). The test verifies the efficacy of the robotic hand in grasping a given object located on top of a table from different approach directions and with different elevation angles with respect to the table. The result is presented in the form of a heat map, which indicates the success rate of grasping from different initial hand poses. For this experiment, we used an apple to test the nominal working condition of the hand and to gain some insight into the influence of possible failures of the hand on the grasping performance. Figure 16 shows the result of the benchmark test if only the force torque sensor (FTS) at the wrist is used to provide feedback. For an approach elevation of zero degrees, the hand can perform the test with a 100% success rate when the FTS is used in combination with the internal proximity sensor of the hand to stop the motion when a contact is detected. Based on this first result, the proximity sensor is also used to detect failures and generate compensatory motions for the subsequent motor failure tests. Note that the hand can also perform the benchmark test on an industrial robot without FTS, as the performance mainly depends on the onboard proximity sensor. For all tests, the hand starts at its zero position, i.e., there was no pregrasp pose of the fingers depending on the scene. For the CLASH hand, the zero position is such that at an approach elevation of zero degrees, the fingers are pointing toward the table.

The first failure we consider is a sensor error of one of the tendon force sensors. We assume that the cable is disconnected, so the pull-up resistor would show the error. If the hand did not react to the failure during a grasp, the high value would trigger the adaptive grasp controller without a contact of one finger to the object, and the result would be an unstable grasp. Figure 16 shows the overall result of grasp performance when the hand uses a simple failure-recovery strategy—namely closing the hand according to the relative position of the object. This position should come from the internal grasp planner, which calculates the motor position for the grasp plus the extra motor motion to get the force calculation based on the characteristic stiffness curves of the hand. The result is poorer than grasping with sensor feedback, which provides a grasp that adapts better to the object shape. This can be seen clearly in the evaluation of the more difficult grasp positions for low approach directions. A more complicated strategy, for example, could use the current sensor to control the grasp force again, but it was not tested in this study due to the additional challenges in its implementation.

On the other hand, higher grasp forces in this simple strategy will also stress the actuators more and would therefore overheat the corresponding actuator. Therefore, a second failure case to be tested is when one of the servos of the fingers is blocked by overheating. For this test, we set a random motor position for the blocked motor and then run the benchmark. We start with the case when the extensor motor of the differential fingers is dead; this motor provides most of the work for stiffness variation. The result can be seen in Figure 16 in the middle row. Due to the design of the differentially coupled distal phalanges, the fingers are equipped with return springs for extension, which separates the motion in the base joint from the motion in the distal joints and helps during a motor failure of the extensor motor to bring the fingers back to the zero position. Therefore, the result is still quite good. With this failure, the fingers lose the capability to change stiffness and compensate for gravity, which is more of a problem for hand orientations of 45 and 135°.

The next motor failure that we tested is in the distal flexors of the differential fingers. The thumb has to press the apple more toward the base phalanges of the fingers. The distal phalanges of the fingers are free and not able to apply forces. The last failure analyzed in this work is a motor failure in one of the flexors of the base. Due to the fixed relative position between apple and hand in the benchmark, a real two-finger grasp is not possible, so the coupled distal flexor has to help the finger with the motor failure to come into contact with the apple. Therefore, the grasp pose is prone to create an undesired contact of the finger with the table in the pre-grasp shape. Furthermore, the performance is very dependent on the hand orientation. If the hand orientation is reversed, the performance will be much worse, because the finger with the failure cannot oppose the force applied by the thumb. Only a linear reorientation of the hand with respect to the apple could increase the performance.

A failure of one of the thumb flexors leads to a simple behavior, as one of the base finger motors fails, but the result is still good, as seen in the corresponding heat map in Figure 16. The grasp performance is clearly not symmetric for the hand orientation angle and would be better if the hand were to be rotated toward the side of the failed flexor. If we now consider a failure of the thumb base extensor, the resulting heat map shows the problem of the missing capability to lift the finger. Furthermore, the thumb might lie in front of the proximity sensor and trigger a false detection signal that prevents proper grasping behavior. A tendon failure at this motor would result in even worse performance at a hand orientation of zero degrees. An extra passive extensor, as in the fingers, would improve the failure behavior but would reduce the positional accuracy if it were not included in the control. Finally, we analyze a motor failure in the thumb distal extensor. Similar to the thumb base extensor, this failure affects the 180° hand orientation grasp performance, as the thumb can more easily come into collision with the apple. The rest of the possible hand orientations still work quite well.

**Figure 17:**
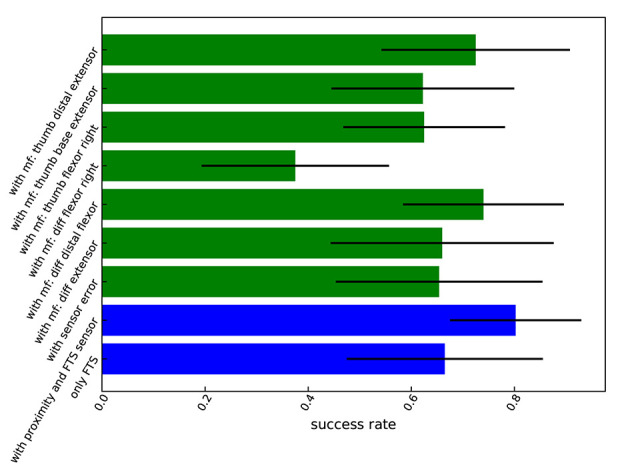
Comparison of grasp performance and variance under motor and sensor failures, plus ground truth performance (two bottom rows: performance with proximity and FTS sensor, or only with FTS sensor).

A comparison of grasp performances for all the analyzed failures and the fully functional hand is presented in Figure 17. Note that some failures, such as failures in the thumb distal extension and finger distal flexor motors, have a very small influence on the grasp success rate for simple objects like an apple, after the grasp pose and force are manually tuned to compensate for the corresponding failure. The grasp pose after the sensor error was not tuned, which results in a larger decrease in success rate.

## 6. Final Discussion

This article summarized the design rationale for the CLASH hand (Compliant Low-Cost Antagonistic Servo Hand) and analyzed the real performance of the hand for its use in real-world scenarios, such as picking up objects in warehouses for online supermarkets. The mechanical robustness of the hand to withstand collisions with the environment was analyzed from a theoretical perspective and studied in an experimental fashion. The accompanying video^3^ presents the sequences of this experimental validation. The hand is able to withstand collisions for impacts on the fingertip in all directions with velocities of up to 4 m/s without any additional action, such as reflex behavior. As a reference, cobots such as Panda (Franka Emika), iiwa (KUKA), and UR5 (Universal Robots) have maximum TCP velocities lower than 2 m/s. For impacts at the finger base, the hand has to react and drive the finger away from the impact. The experiments showed that the best strategy for minimizing the effect of an unforeseen impact is to activate the admittance control of the hand, which reduces the loads and also allows higher robot velocities while still guaranteeing the safety of the hand. Higher passive compliance of the hand can still be achieved by increasing the spring deflection of the FAS if the application scenario requires this. It is also clear that when increasing the stiffness of the hand, the robot has to move slower to protect the hand; however, for robot motions without a grasped object, it is not necessary to increase the stiffness. If the finger must be stiff to transmit forces, the robot needs to move slower to guarantee proper performance even in the presence of unexpected collisions.

Furthermore, we looked into the capability of the CLASH hand to cope with sensor and motor failures, for which we implemented a self-check of the hand that provides feedback to the grasp controller in case of an error showing up. The hand performance (ground-truth) was tested with a benchmark developed specifically for analyzing the performance of soft-end effectors. Artificial errors were induced in the sensors and motors to compare the ground-truth performance with the performance when error-adaption strategies are implemented. For a simple object like an apple, the top grasp performance is still 100% successful, which shows that differentially coupled joints and independently actuated fingers can outperform underactuated hands because motor or transmission failures stop a finger or the whole hand in the latter case. Furthermore, we implemented observers for grasp success and to verify whether the object is lost, which can help artificial intelligence systems in the future to learn better grasping strategies without requiring that a human manually labels the success of the grasp. Such an observer can also start a reactive strategy to keep the object grasped within the hand, which proved useful in the studied failure cases.

## Data Availability Statement

The datasets generated for this study are available on request to the corresponding author.

## Author Contributions

WF developed the robotic hand, leading the mechatronic design, and contributed to the literature review, design and execution of experiments, data analysis, and paper writing. MR contributed to the hand design, interpretation of experiments, and paper writing.

### Conflict of Interest

The authors declare that the research was conducted in the absence of any commercial or financial relationships that could be construed as a potential conflict of interest.

## Footnotes

^1^The SoMa (SOft MAnipulation) project, http://soma-project.eu/, aimed to use soft robotic solutions that allow contact-rich interactions with the environment to grasp delicate objects from relatively cluttered and geometrically constrained setups. It included a fruit handling use case provided by Ocado, the world's largest online-only supermarket.

^2^https://youtu.be/6sLet9blWdA

^3^https://youtu.be/IfZVJ8Iv4Qg
